# Potential of Immune-Related Genes as Biomarkers for Diagnosis and Subtype Classification of Preeclampsia

**DOI:** 10.3389/fgene.2020.579709

**Published:** 2020-12-01

**Authors:** Ying Wang, Zhen Li, Guiyu Song, Jun Wang

**Affiliations:** Department of Obstetrics and Gynecology, Shengjing Hospital of China Medical University, Shenyang, China

**Keywords:** preeclampsia, immunity, biomarker, nomogram, consensus clustering

## Abstract

**Objective:**

Preeclampsia is the main cause of maternal mortality due to a lack of diagnostic biomarkers and effective prevention and treatment. The immune system plays an important role in the occurrence and development of preeclampsia. This research aimed to identify significant immune-related genes to predict preeclampsia and possible prevention and control methods.

**Methods:**

Differential expression analysis between normotensive and PE pregnancies was performed to identify significantly changed immune-related genes. Generalized linear model (GLM), random forest (RF), and support vector machine (SVM) models were established separately to screen the most suitable biomarkers for the diagnosis of PE among these significantly changed immune-related genes. The consensus clustering method was used to divide the PE cases into several subgroups to explore the function of the significantly changed immune-related genes in PE.

**Results:**

Thirteen significantly changed immune-related genes were obtained by the differential expression analysis. RF was the best model and was used to select the four most important explanatory variables (CRH, PI3, CCL18, and CCL2) to diagnose PE. A nomogram model was constructed to predict PE based on these four variables. The decision curve analysis (DCA) and clinical impact curves revealed that PE patients could significantly benefit from this nomogram. Consensus clustering analysis of the 13 differentially expressed immune-related genes (DIRGs) was used to identify 3 subgroups of PE pregnancies with different clinical outcomes and immune cell infiltration.

**Conclusion:**

Our study identified four immune-related genes to predict PE and three subgroups of PE with different clinical outcomes and immune cell infiltration. Future studies on the three subgroups may provide direction for individualized treatment of PE patients.

## Introduction

Preeclampsia (PE) is an idiopathic disease occurring during pregnancy, which can affect the function of multiple organs (Grandi et al., 2019; Phipps et al., 2019). PE affects 3–5% of pregnant women worldwide (Zhang et al., 2020b). It is a hypertension disease that is one of the main causes of increased mortality for pregnant women and perinatal infants (Say et al., 2014). In the absence of effective medical intervention, PE may progress to eclampsia, so effective diagnosis and treatment are crucial.

The etiology and pathogenesis of PE are complex and have not been fully elucidated. In recent years, an imbalance in the immune system has been closely related to PE occurrence and development (Ma et al., 2019). A placental origin for PE is widely accepted. In early pregnancy, successful placental implantation depends on the precise regulation of the maternal immune system (Redman and Sargent, 2010; LaMarca et al., 2013). During this process, immune cells are needed for the invasive behavior observed in the decidual layer of the uterus. These immune cells gather around the trophoblasts and carry out different functions. They can control the migration of *in situ* cells of the spiral arteries and make trophoblasts migrate moderately to the intima by secreting cytokines and angiogenic factors (Perez-Sepulveda et al., 2014; Rahimzadeh et al., 2016). The balance between the immune cells and cytokines produced at the maternal-fetal interface is important for a normal pregnancy. Any local imbalance in the immune response may lead to abnormal placental structure or angiogenesis (Robillard et al., 2014; Sahay et al., 2014; LaMarca et al., 2016). Indeed, an insufficient invasion of trophoblasts during placental implantation leads to defective placental spiral artery remodeling, and both placenta and fetus are in a relatively ischemic state (Lai et al., 2020).

In our research, we screened for differentially expressed immune-related genes (DIRGs) between normotensive and PE pregnancies in the GSE60438 dataset. A stochastic forest model was constructed to predict the potential value of the DIRGs in diagnosing PE, and the important DIRGs were selected to establish a nomogram model to predict PE. In addition, we used a consensus clustering algorithm to classify PE pregnancies into three subgroups based on DIRG expression and explored the immune phenotype of these three subgroups.

## Materials and Methods

### Data Source

The GSE60438 dataset containing two batches was obtained from the GEO database^1^. The second batch including 42 normotensive and 35 PE pregnancies was selected as training dataset (Yong et al., 2015). The first batch including 23 normotensive and 25 PE pregnancies was selected as validation dataset. We downloaded the gene expression profile and clinical data for our research. The probe number of the expression profile data was converted to gene symbols based on the annotation files. We obtained 2,499 immune-related genes (IRGs) from the ImmPort database^2^. The IRGs contained genes related to antigen processing and presentation, antimicrobials, the BCR and TCR signaling pathways, chemokines, cytokines, interleukins, and their respective receptors, natural killer cell cytotoxicity, and TGFb, TGFb receptor, TNF, and TNF receptor family members (Bhattacharya et al., 2014).

### Screening for Differentially Expressed Immune-Related Genes

The “limma” package in R was used to identify differentially expressed genes (DEGs) between normotensive and PE pregnancies. We then crossed the DEGs with the IRGs to obtain the DIRGs for further investigation.

### Construction and RF, GLM, and SVM Models

We created generalized linear (GLM), random forest (RF), and support vector machine (SVM) models based on the training set. Positive-negative class balancing has been done by Combination/integration approach. The occurrence or absence of PE was used as the response variable, and the DIRGs were used as the explanatory variables. Next, we used the explain function of the “DALEX” package in R to analyze the three models and plotted the residual distribution to select the best model based on the validation dataset. Finally, we analyzed the importance of the variables and selected the four most important explanatory variables for further study.

### Construction and Evaluation of the Nomogram Model

A nomogram model was constructed using the “rms” package to facilitate the clinical application. The “Points” indicate the score of each factor under different conditions, while the “Total Points” refer to the total score of all factors. We measured the predictive accuracy of the nomogram by calibration curves and generated a clinical impact curve. The decision curve analysis (DCA) data were plotted to evaluate the clinical value of the nomogram.

### Consensus Clustering for DEGs

We performed consensus clustering to divide the PE cases into several subgroups based on the expression profiles of the identified DEGs using the “ConsensusClusterPlus” package in R. We used 1,000 iterations to ensure the stability of the clustering. The consensus k number, which was used to select the number of subgroups to divide the PE samples into, was determined by the cumulative distribution function (CDF) curves, delta area score of CDF, and consensus matrix heat maps (Wilkerson and Hayes, 2010; Zhang et al., 2020a).

### Evaluation of the Proportion of 22 Immune Cell Types in PE Pregnancies

Currently, the algorithms used to estimate the cell components in tissue based on gene expression profiles can be divided into two categories: Gene Set Enrichment Analysis (GSEA) and deconvolution. CIBERSORT is a deconvolution algorithm that can combine the labeled genomes of different immune cell subpopulations to calculate the proportion of 22 immune cell types in tissues. These immune cell types are presented in Supplementary Table 1. In this study, the CIBERSORT online platform^3^ was used for this analysis, and each sample obtained a *p*-value. Samples with a CIBERSORT output value of *p* < 0.05 were considered statistically significant and analyzed further (Zhou et al., 2018; Zhang et al., 2019).

### Statistical Analysis

We used Wilcox.test or Kruskal-Wallis tests to compare the differences between the groups. The “RCircos” package in R was used to map the chromosomal positions of the DIRGs. Spearman correlation analysis was performed to calculate the correlation coefficients between the DIRGs. A two-sided *p*-value of less than 0.05 was considered statistically significant. All statistical analyses were performed using R 4.0.0.

## Results

### Landscape of DIRGs in PE Pregnancies

**FIGURE 1:**
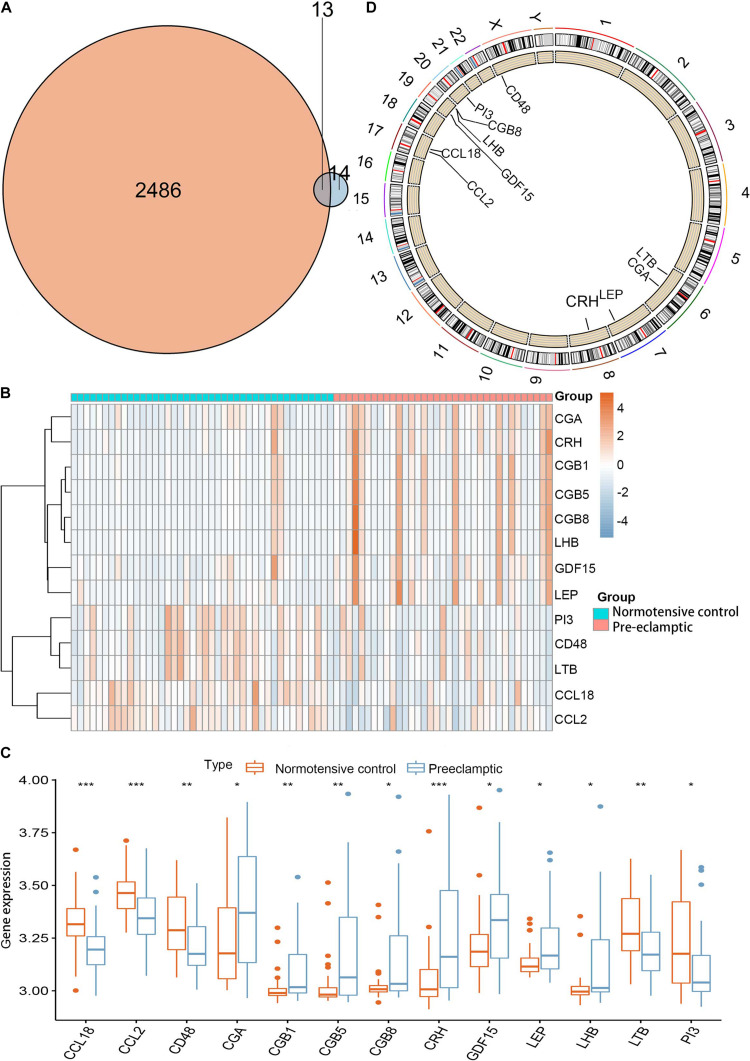
Landscape of 13 DIRGs in PE pregnancies. **(A)** The Venn diagram shows the identification of 13 DIRGs by crossing the 27 DEGs with 2,499 IRGs. **(B)** The expression heat map of the 13 DIRGs in normotensive and PE pregnancies. **(C)** The differential expression histogram of the 13 DIRGs identified between the normotensive and PE pregnancies. **(D)** The chromosomal positions of the 13 DIRGs. *P < 0.05; **P < 0.01; ***P < 0.001.

**FIGURE 2:**
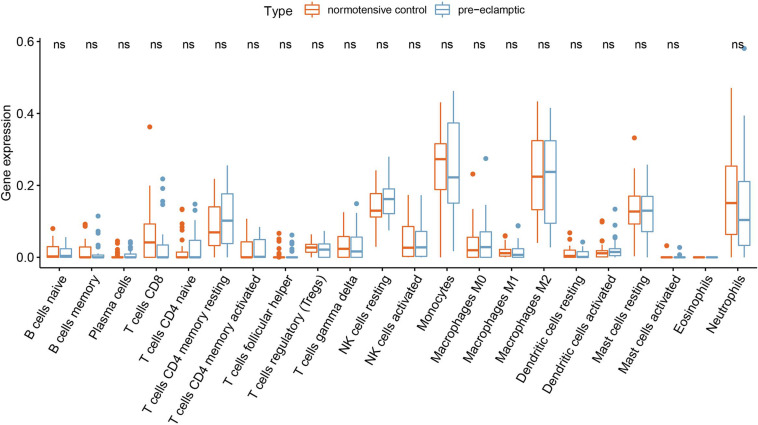
Differential immune cell infiltration between normotensive and PE pregnancies.

Twenty-seven DEGs were identified between normotensive and PE pregnancies according to the screening criteria of log | FC| > 0.1 and *p* < 0.05 using the “limma” package based on the training dataset. We obtained 13 DIRGs by crossing the 27 DEGs with 2,499 IRGs, which were used to generate the Venn diagram (Figure 1A). In addition, 36 immune genes in our analysis overlapped with the original article according to the screening criteria of *P* < 0.05. The overlapping immune genes were listed in Supplementary Table 1. The expression of the 13 DIRGs in the normotensive and PE pregnancies are shown in Figures 1B,C. Both the heat map and the histogram showed that PI3, CCL18, CCL2, LTB, and CD48 were expressed at low levels in PE pregnancies compared to normotensive pregnancies. In contrast, the expression levels of LEP, CGB1, CDF15, LHB, CGB8, CGA, CGB5, and CRH were higher in the PE pregnancies than in the normotensive pregnancies. The chromosomal positions of the 13 DIRGs are shown in Figure 1D. We compared the immune infiltration between normotensive and PE pregnancies. The results revealed that the immune infiltration between normotensive and PE pregnancies have no statistical differences (Figure 2). It is generally known that PE pregnancies can cause premature birth at a gestational age of fewer than 37 weeks or an infant weight of less than 2.5 kg. This phenomenon is presented in Supplementary Figures 1A,B. Thus, we also investigated the relationship between the 13 DIRGs and clinical information (e.g., gestational age and infant weight). The results indicated that the 13 DIRGs were associated with premature birth (Supplementary Figures 1C,D).

### Construction and RF Model

**FIGURE 3:**
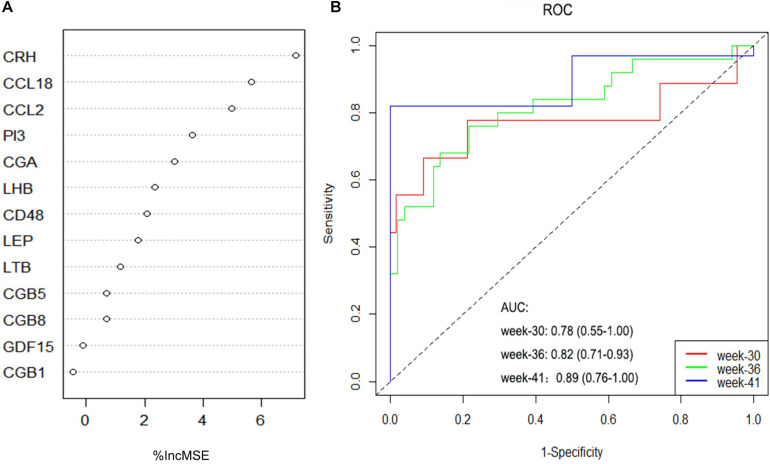
Construction and evaluation of the RF models. **(A)** The importance of the variables based on the RF model at the gestational age from 25 to 41 week. **(B)** ROC curves indicated the accuracy of the RF model based on the four most important explanatory variables at 30-, 36-, and 41-week.

**FIGURE 4:**
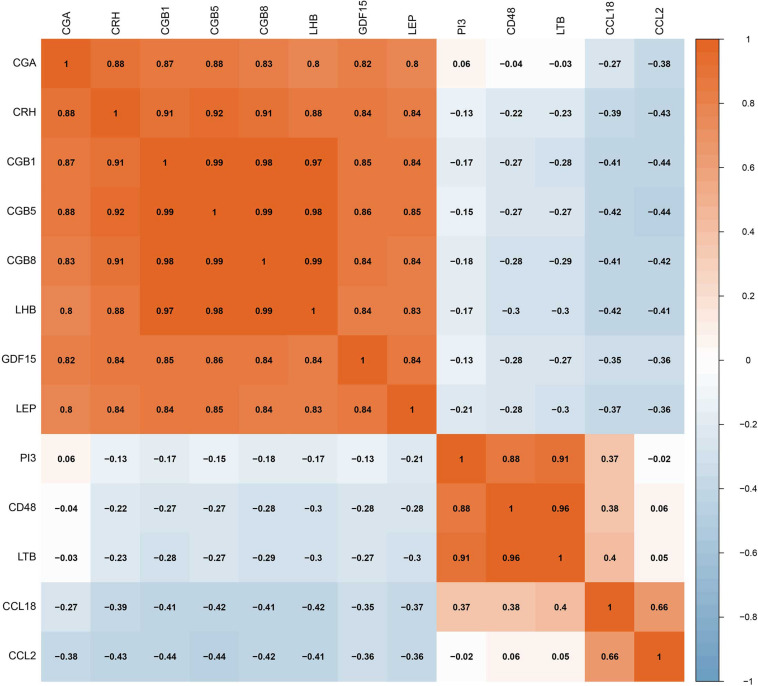
The correlation coefficients between the 13 DIRGs by Spearman correlation analysis.

The RF, GLM, and SVM models were each established using the training set. We explained the three models using the “DALEX” package and plotted the residual distribution to select the best model based on the test set. The RF model had the least sample residual (Supplementary Figure 2). Thus, the RF model was constructed to distinguish normotensive and PE pregnancies in our research. We analyzed the importance of the variables based on the RF model at the gestational age from 25 to 41 week (Figure 3A). Variable importance is ranked according to %IncMSE. “%IncMSE” represents an increase in mean squared error. The more important a predictor gene is, the greater the prediction error will be, when its value is randomly replaced. Then, we performed a 10-fold cross validation to select the appropriate important variables and four most important explanatory variables (CRH, PI3, CCL18, and CCL2) from the RF model was selected for further evaluation. We calculated the risk score of each patients based on the regression coefficients using multivariate Cox proportional hazards regression (PHR) analysis. The risk score of each patients in training dataset was present in Supplementary Table 2. The time-dependent ROC curves indicated that the accuracy of the RF model is well based on the four most important explanatory variables at 30-, 36-, and 41-week (Figure 3B). The AUC value of the ROC curve increased with the elevated gestational weeks. The four most important explanatory variables have the most predictive power at 41-week during pregnancy. Correlation analysis of the 13 DIRGs revealed that the correlation coefficients between the four selected DIRGs were lower (Figure 4), suggesting the RF model exclude genes with similar functions to simplify the model and reduce unnecessary costs.

### Construction and Evaluation of the Nomogram Model

**FIGURE 5:**
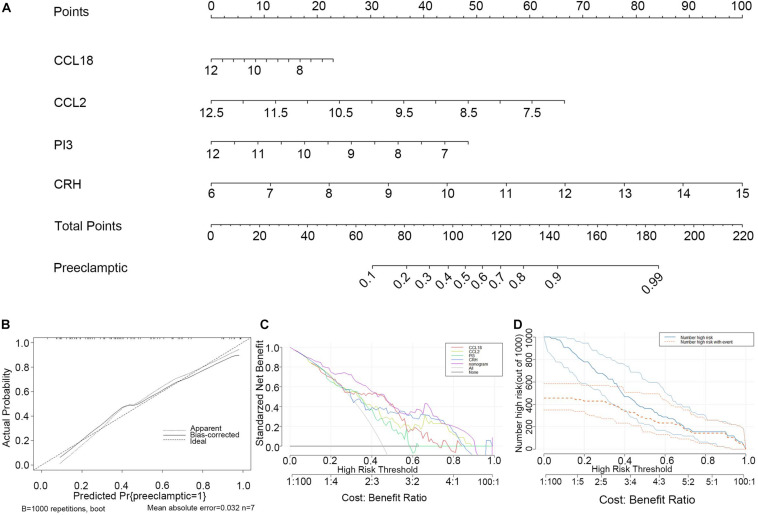
Construction and evaluation of the nomogram model based on the four explanatory variables from training dataset. **(A)** Construction and evaluation of the nomogram model based on the four explanatory variables. **(B)** The calibration curve revealed the predictiveness of the nomogram model. **(C)** The DCA curve evaluated the clinical value of the nomogram model. **(D)** The clinical impact curve used to assess the clinical impact of the nomogram model.

To facilitate the ability to predict PE pregnancies clinically, we constructed a nomogram model using the “rms” package based on the four selected DIRGs (CRH, PI3, CCL18, and CCL2) from the training dataset (Figure 5A). Calibration curves revealed that the predictiveness of the nomogram model was accurate (Figure 5B). The DCA curve was plotted to evaluate the clinical value of this nomogram model. The x-axis indicates the predicted probability, and the y-axis represents the net benefit. The oblique red line revealed that the nomogram model could benefit patients at high risk threshold from 0.1 to 0.9 (Figure 5C). We further evaluated the clinical impact curve based on the DCA curve to more intuitively assess the clinical impact of this model. We found that the predictive power of the nomogram model was remarkable. The predicted number of high-risk patients was greater than that of high-risk patients with an event (Figure 5D). We then test the nomogram model in the validation dataset based on the four selected DIRGs. Calibration curves revealed that the accuracy of the nomogram model was good (Supplementary Figure 3A). DCA curve and clinical impact curve indicated that patients can benefit from the nomogram at high risk threshold almost from 0.1 to 0.9 (Supplementary Figures 3B,C).

### Three Subgroups Obtained by Consensus Clustering

**FIGURE 6:**
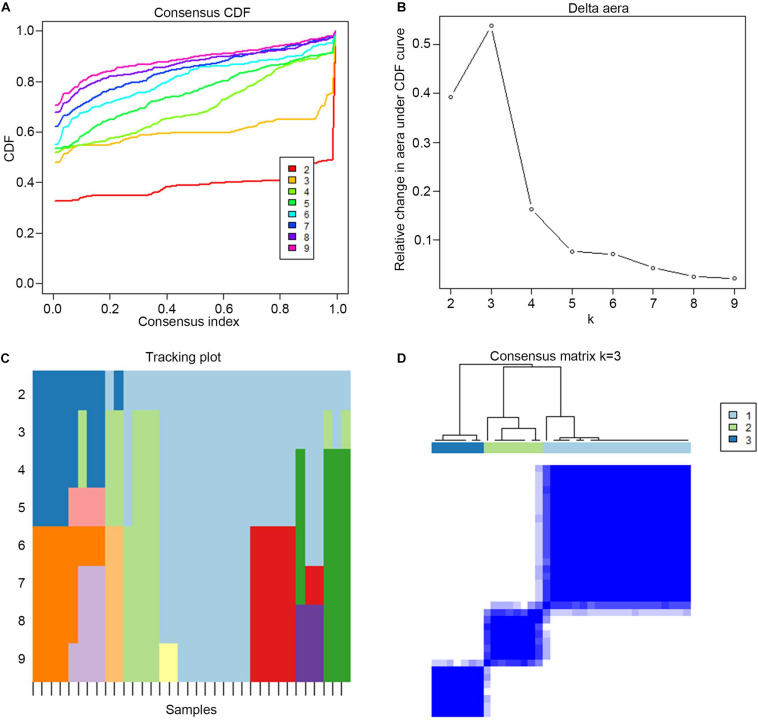
The three subgroups identified by consensus clustering based on the 13 DEGs from training dataset. **(A)** CDF curve for *k* = 2–9. It shows the cumulative distribution function of different k values, which can be used to determine when k is taken, the CDF reaches an approximate maximum value, and the clustering analysis results are the most reliable. **(B)** The delta area score of the CDF curve from *k* = 2–9. It shows the relative change of area under the CDF curve. **(C)** Tracking plot from *k* = 2–9. The black stripe at the bottom represents the sample, showing the classification of the sample when different values of k are taken, and the color blocks of different colors represent different classifications. **(D)** The matrix heat map were clearly separated when *k* = 3. The rows and columns of the matrix heat map represent samples. The values of the consistency matrix are white to dark blue from 0 (impossible to cluster together) to 1 (always clustered together). The consistency matrix is arranged according to the consistency classification (the tree above the heat map). The bar between the tree view and the heat map is the classification.

**FIGURE 7:**
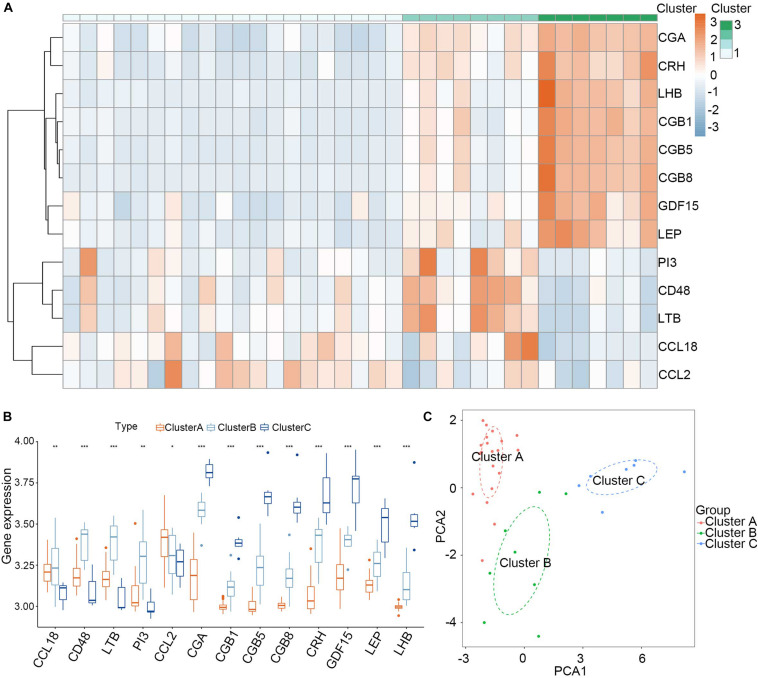
Landscape of three subgroups in PE pregnancies in training dataset. **(A)** The expression heat map of 13 DIRGs between the three subgroups. **(B)** The differential expression histogram of 13 DIRGs between the three subgroups. **(C)** Principal component analysis for the expression profiles of three subgroups that shows a remarkable difference in the transcriptomes between the different subgroups. *P < 0.05; **P < 0.01; ***P < 0.001.

Consensus clustering was used to divide the PE cases into several subgroups to explore the function of the 13 DEGs in PE based on their expression profiles. We plotted a CDF curve that allows us to determine at the k number. The CDF can have an approximate maximum when we choose the most suitable *k* = 3 (Figure 6A). Figure 6B shows the delta area score of the CDF curve from *k* = 2–9. We found that the relative increase in the delta area score tends to be stable after *k* = 3. Thus, the PE cases could be divided into three subgroups. Figure 6C provides a view of the item cluster membership across the different k numbers to track the cluster history. The matrix heat maps were clearly separated when *k* = 3 (Figure 6D). We also explored the expression of the 13 DIRGs in the three subgroups (clusters A, B, and C). The heat map and the histogram showed that CCL18, CD48, LTB, and PI3 were highly expressed in cluster B, whereas CCL2 was highly expressed in cluster A, and CGA, CGB1, CGB5, CGB8, CRH, GDF15, LEP, and LHB were highly expressed in cluster C compared to the other subgroups (Figures 7A,B). The principal component analysis (PCA) indicated that the expression of the 13 DIRGs could completely distinguish the three subgroups (Figure 7C).

We then test the consensus clustering in validation dataset based on the 12 DEGs (GDF15 gene could not be found in the validation dataset) and found similar grouping results (Supplementary Figure 4). The heat map and the histogram revealed that except for CCL2 gene, the difference distribution of the other genes in the three groups was similar (Supplementary Figures 5A,B). The principal component analysis (PCA) indicated that the expression of the 12 DIRGs could also distinguish the three subgroups (Supplementary Figure 5C).

### Clinical Traits and Immune Cell Infiltration Characteristics of the Three Subgroups

**FIGURE 8:**
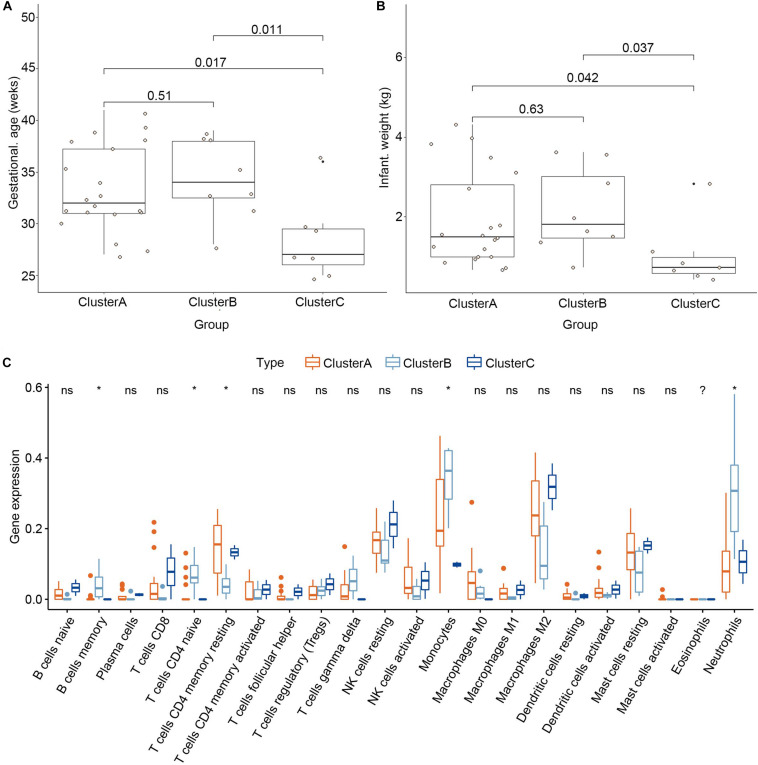
Clinical traits and immune cell infiltration characteristics of the three subgroups. **(A)** Differential gestational age between the three subgroups. **(B)** Differential infant weight between the three subgroups. **(C)** Differential immune cell infiltration between the three subgroups. *P < 0.05.

To explore the significance of the three subgroups, we compared the gestational age and infant weight between the three subgroups by Kruskal-Wallis tests based on the training dataset. We found that the cluster C subgroup had a lower gestational age and infant weight than the other two subgroups (Figures 8A,B). Differential analysis of the immune cell infiltration levels in the three subgroups revealed that infiltration by the memory B cells, naïve CD4^+^ T cells, γδ T cells, monocytes, and neutrophils in the cluster B subgroup was higher than in the other subgroups while resting dendritic cells and M2 macrophages were found in higher numbers in the cluster C subgroup (Figure 8C).

## Discussion

PE is the main cause of maternal mortality (Abalos et al., 2013). There is currently no gold standard for diagnosis or effective preventive and treatment methods. The immune system consists of cytokines, chemokines, inhibitory receptors, and ligands, and immune cells that play important roles in normal pregnancy (Kubes and Jenne, 2018). An immune imbalance may lead to pregnancy-specific complications, including PE (Schumacher et al., 2018). In our research, we explored the significance of immune-related genes in PE and established a basis for screening diagnosis markers and individualized immunotherapy for PE.

We screened 13 immune-related genes (PI3, CCL18, CCL2, LTB, CD48, LEP, CGB1, CDF15, LHB, CGB8, CGA, CGB5, and CRH) based on significant differential expression analysis between normotensive and PE pregnancies. PI3, CCL18, CCL2, LTB, and CD48 were downregulated in PE. In contrast, LEP, CGB1, CDF15, LHB, CGB8, CGA, CGB5, and CRH were upregulated. Most of the significant immune-related genes belonged to the category of antimicrobials and cytokines that function in the reaction to bacteria, inflammatory responses, and immune processes. We also explored the relationship between the 13 DIRGs and premature birth. The results suggested that low expression of PI3, CCL18, CCL2, LTB, and CD48 and high expression of LEP, CGB1, CDF15, LHB, CGB8, CGA, CGB5, and CRH might be involved in the occurrence of premature delivery. These data are consistent with the adverse outcome of premature delivery caused by PE. RF is an integrated algorithm composed of a decision tree, which can be used as a classification tool. The decision tree produces a tree model, classifies the data, and forecasts by repeatedly distinguishing each variable. RF has numerous merits, such as judging the importance of features, does not easily overfit, and its training speed is relatively fast (Jeong et al., 2020). GLM is a widely used statistical model that is mainly used to solve a binary classification problem, and represents the possibility of something happening (Qu and Luo, 2015). SVM was first proposed by Cortes and Vapnik in 1963 (Ben-Dor et al., 2000). It has many unique advantages in solving small sample, nonlinear, and high-dimensional pattern recognition (Huang et al., 2018). We constructed different models based on these methods and found that the RF model was the best model for identifying the most suitable biomarkers for diagnosing PE. Indeed, the DCA and clinical impact curves demonstrated that PE patients could benefit from the nomogram generated using the four explanatory variables (CRH, PI3, CCL18, and CCL2) identified using the RF model in both training dataset and texting dataset. We used consensus clustering analysis of the 13 DIRGs to classify PE pregnancies into three subgroups. CCL2 was higher expression in cluster A subgroup compared with the other groups, while CCL18, CD48, LTB, PI3 were higher expression in cluster B subgroup, and CGA, CGB1, CGB5, CGB8, CRH, CDF15, LEP, LHB were higher expression in cluster C subgroup. We also found similar grouping results in texting dataset based on the 12 DEGs (GDF15 gene could not be found in the validation dataset). The heat map and the histogram revealed that except for CCL2 gene, the difference distribution of the other genes in the three groups was similar, which revealed the stability of the grouping by consensus clustering analysis.

Proteins related to the genes higher expression in cluster C subgroup were mainly hormone-associated protein and the cluster C subgroup had a lower gestational age and infant weight than the other groups. Therefore, boldly speculate that a large number of hormone levels are positively correlated with the risk of PE and the conjecture was highly consistent with previous studies (Makrigiannakis et al., 2018; Al-Kaabi et al., 2020; Daskalakis et al., 2020). Antonis Makrigiannakis suggested that corticotropin releasing hormone (CRH) may contribute to preeclampsia (Makrigiannakis et al., 2018). The β-hCG encoded by CGB5 and CGB8 genes was demonstrated positive correlation with preeclampsia (Al-Kaabi et al., 2020). Georgios Daskalakis reported that preeclampsia is associated with increased leptin (LEP) (Daskalakis et al., 2020). In addition, we found that memory B cells, naïve CD4+T cells, γδ T cells, monocytes, and neutrophils had higher infiltration in the cluster B subgroup. Similarly, resting dendritic cells and M2 macrophages showed higher infiltration in the cluster C group. We speculate that immunosuppression may cause a disorder in the internal environment, leading to premature birth. Additional research is needed to explore the relationship between immune cell infiltration and the PE subgroups, gestational age, and infant weight of the subgroups of PE patients.

This study had some limitations. Firstly, the sample tissues came from decidual tissue rather than blood, so we could not determine whether the selected diagnostic markers would be suitable for measuring in blood samples. Secondly, our research results are only predictions. These results need to be verified by basic science and clinical studies. Thirdly, since the decidual basalis samples are taken at the occurrence of PE, it will be interesting to see whether the biomarkers cytokines can be detected in serum. However, we searched the data sets in the database and found no transcriptome data that met the requirements. Fourthly, we did not verify our research results using other datasets due to the limitations of the data in the database. Finally, the key immune-related genes used for PE diagnosis were selected based on the placental tissue samples at the gestational age from 25 to 41 week. Whether these genes have the potential to be biomarkers for early diagnosis need further experimental studies.

## Conclusion

In conclusion, our study selected four explanatory variables and established a nomogram model to predict PE. We also identified three subgroups with different clinical outcomes and immune cell infiltration. Future studies on these three subgroups may provide direction for individualized treatment of PE patients.

## Data Availability Statement

The datasets generated for this study can be found in the online repositories. The names of the repository/repositories and accession number(s) can be found in the article/ Supplementary Material.

## Author Contributions

All authors listed have made a substantial, direct and intellectual contribution to the work, and approved it for publication.

## Conflict of Interest

The authors declare that the research was conducted in the absence of any commercial or financial relationships that could be construed as a potential conflict of interest.

## Footnotes

1 http://www.ncbi.nlm.nih.gov/geo

2 https://immport.niaid.nih.gov

3 http://cibersort.stanford.edu/

## Supplementary Material

Supplementary Figure 1 Correlations between the clinicopathological features and the 13 DIRGs. **(A)** Differential gestational age between normotensive and PE pregnancies. **(B)** Differential infant weight between normotensive and PE pregnancies. **(C)** The differential expression of the 13 DIRGs among distinct gestational ages. **(D)** The differential expression of the 13 DIRGs among distinct infant weights.

Supplementary Figure 2 Residual distributions of the RF, GLM, and SVM models.

Supplementary Figure 3 Construction and evaluation of the nomogram model based on the four explanatory variables from validation dataset. **(A)** Construction and evaluation of the nomogram model based on the four explanatory variables. **(B)** The calibration curve revealed the predictiveness of the nomogram model. **(C)** The DCA curve evaluated the clinical value of the nomogram model. **(D)** The clinical impact curve used to assess the clinical impact of the nomogram model.

Supplementary Figure 4 The three subgroups identified by consensus clustering based on the 12 DEGs from validation dataset.

Supplementary Figure 5 Landscape of three subgroups in PE pregnancies in validation dataset. **(A)** The expression heat map of 12 DIRGs between the three subgroups. **(B)** The differential expression histogram of 12 DIRGs between the three subgroups. **(C)** Principal component analysis for the expression profiles of three subgroups that shows a remarkable difference in the transcriptomes between the different subgroups.

Supplementary Table 1 Thirty-six immune genes in our analysis overlapped with the original article according to the screening criteria of *P* < 0.05.

Supplementary Table 2 The risk score of each patients in training dataset.
