# Supplementary figures and images for: Potential of Immune-Related Genes as Biomarkers for Diagnosis and Subtype Classification of Preeclampsia
Source: Front Genet. 2020 Dec 1;11:579709. doi: 10.3389/fgene.2020.579709 (PMC7737719; doi:10.3389/fgene.2020.579709)

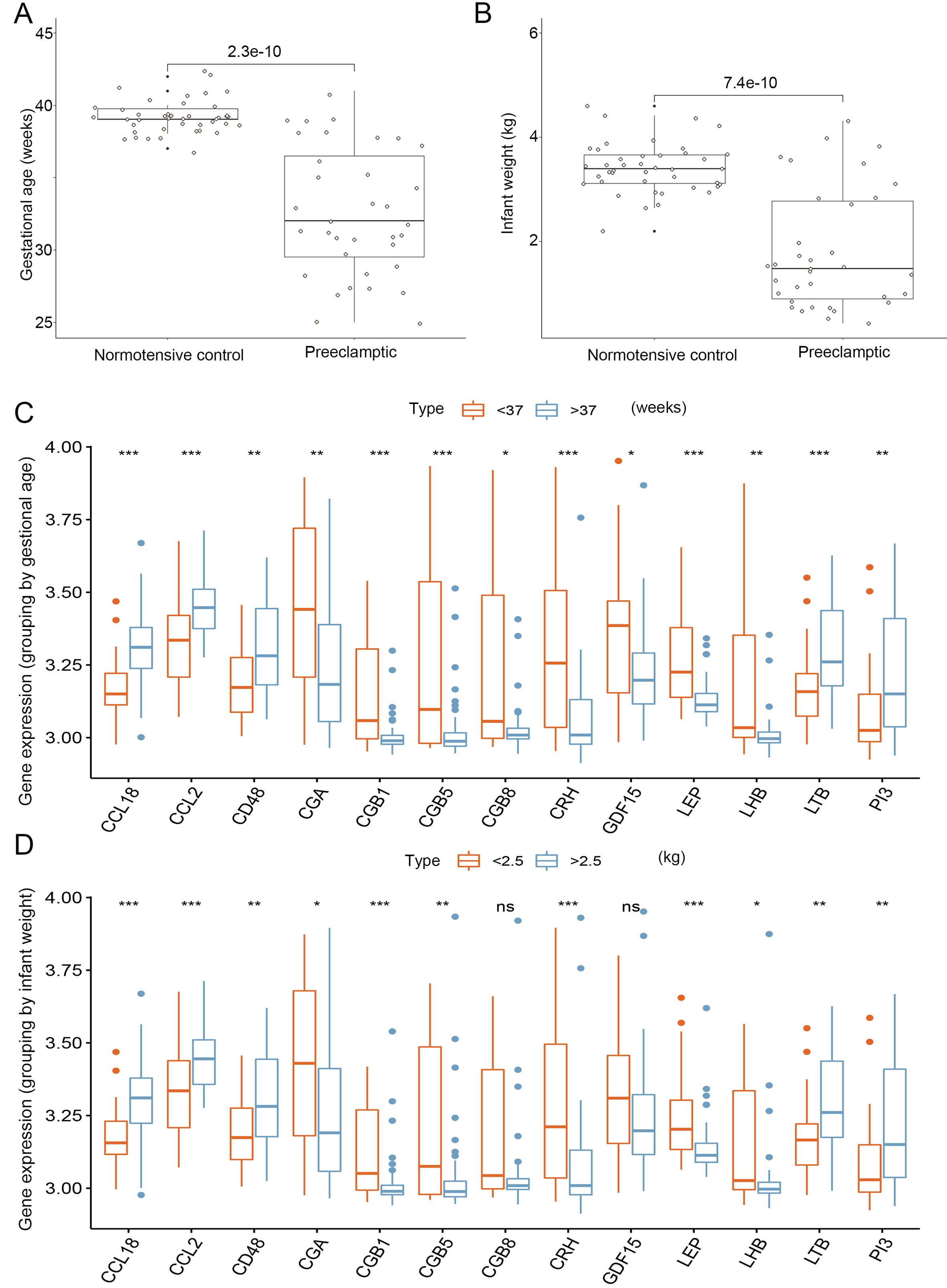

Supplement: Supplementary Figure 1 — Correlations between the clinicopathological features and the 13 DIRGs. (A) Differential gestational age between normotensive and PE pregnancies. (B) Differential infant weight between normotensive and PE pregnancies. (C) The differential expression of the 13 DIRGs among distinct gestational ages. (D) The differential expression of the 13 DIRGs among distinct infant weights. [file Image_1.jpeg]

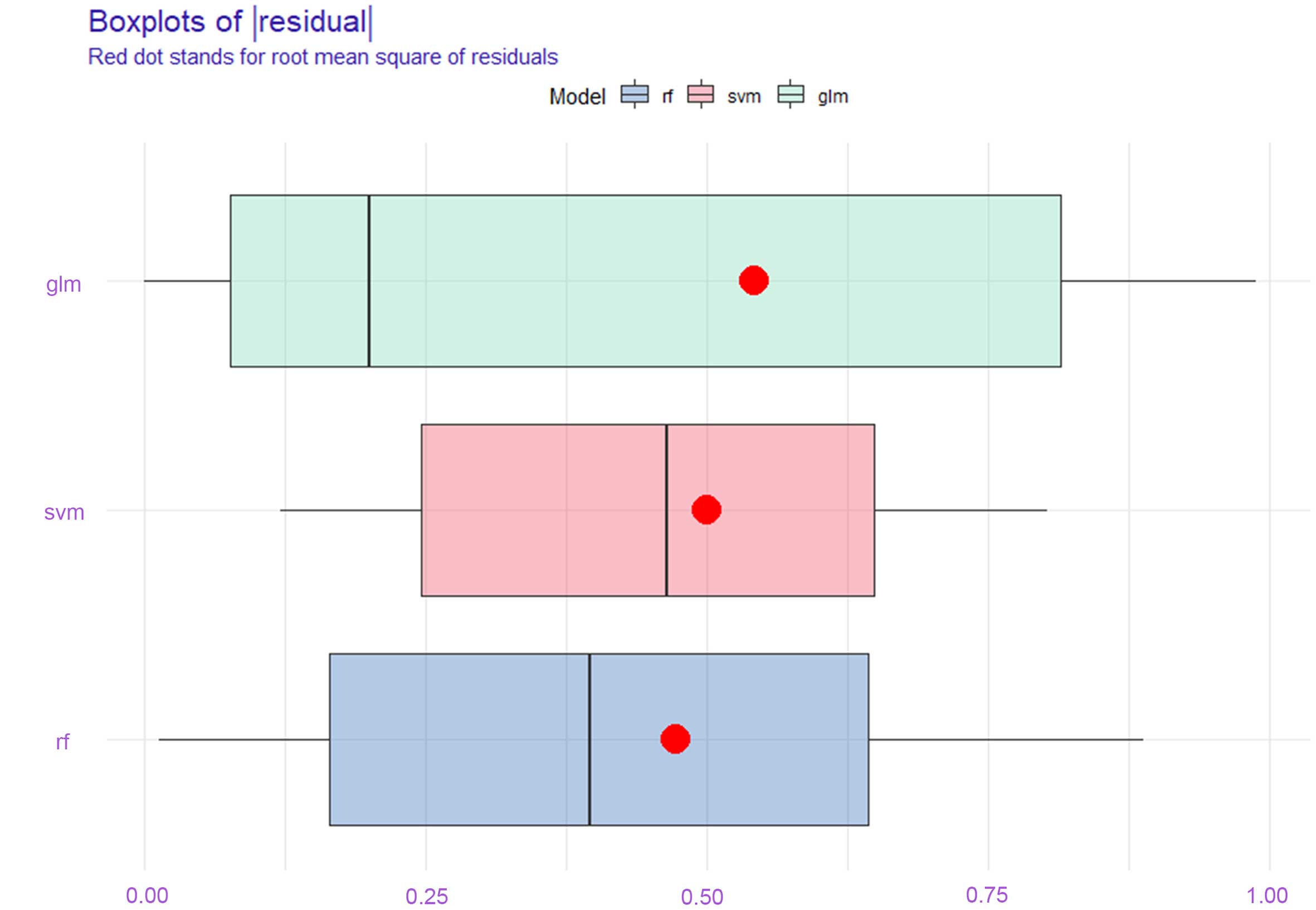

Supplement: Supplementary Figure 2 — Residual distributions of the RF, GLM, and SVM models. [file Image_2.jpeg]

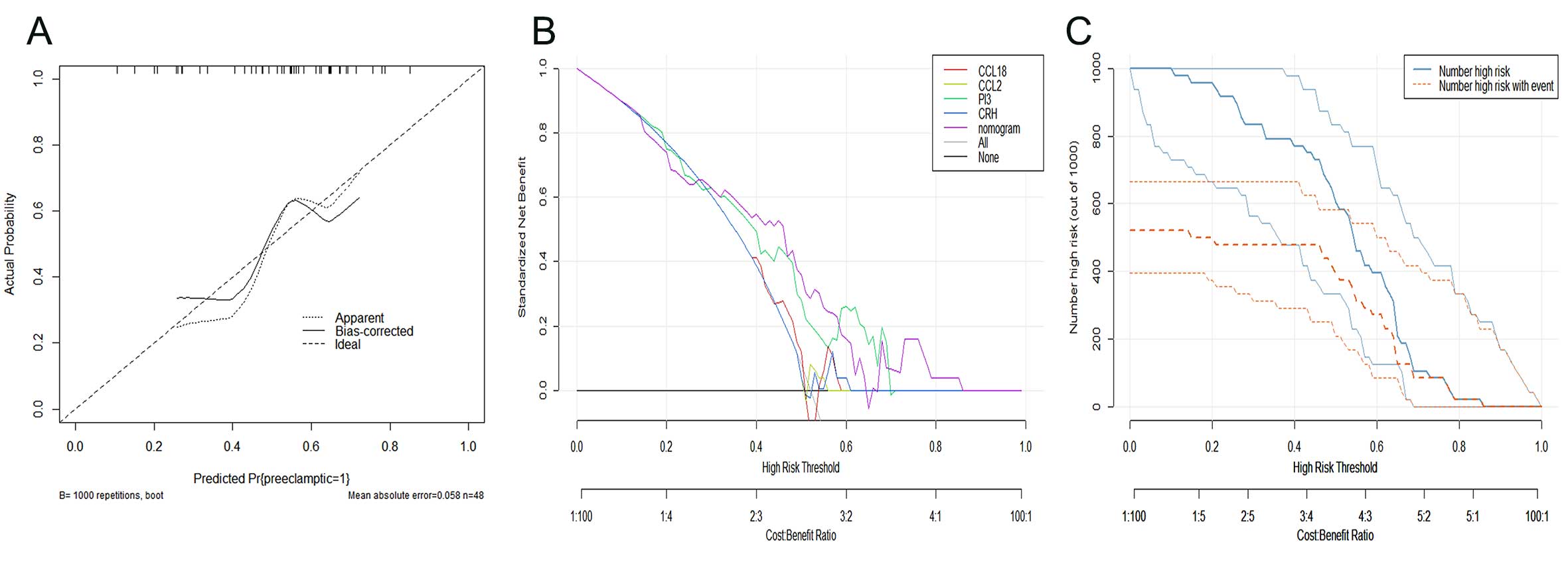

Supplement: Supplementary Figure 3 — Construction and evaluation of the nomogram model based on the four explanatory variables from validation dataset. (A) Construction and evaluation of the nomogram model based on the four explanatory variables. (B) The calibration curve revealed the predictiveness of the nomogram model. (C) The DCA curve evaluated the clinical value of the nomogram model. (D) The clinical impact curve used to assess the clinical impact of the nomogram model. [file Image_3.jpeg]

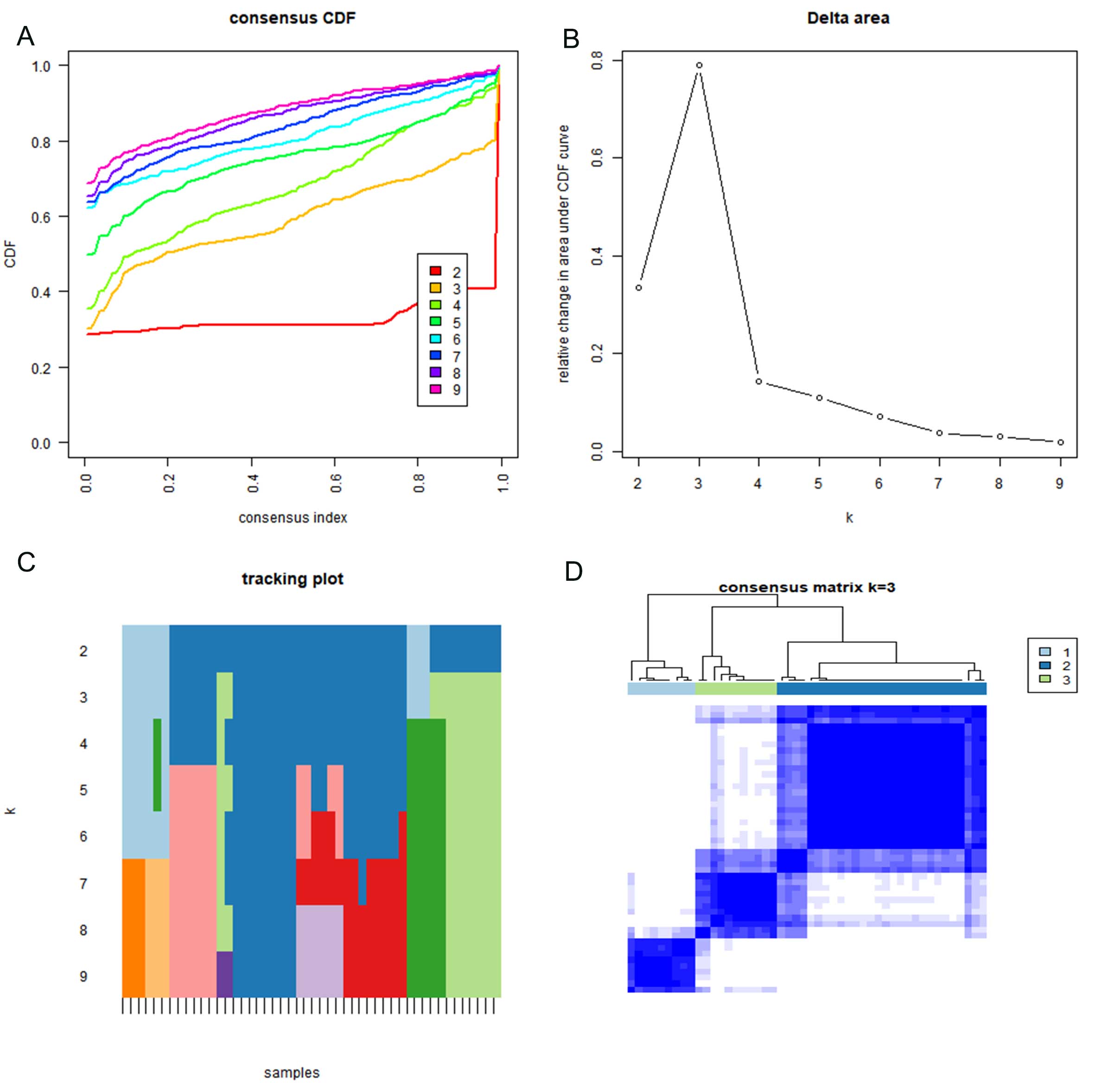

Supplement: Supplementary Figure 4 — The three subgroups identified by consensus clustering based on the 12 DEGs from validation dataset. [file Image_4.jpeg]

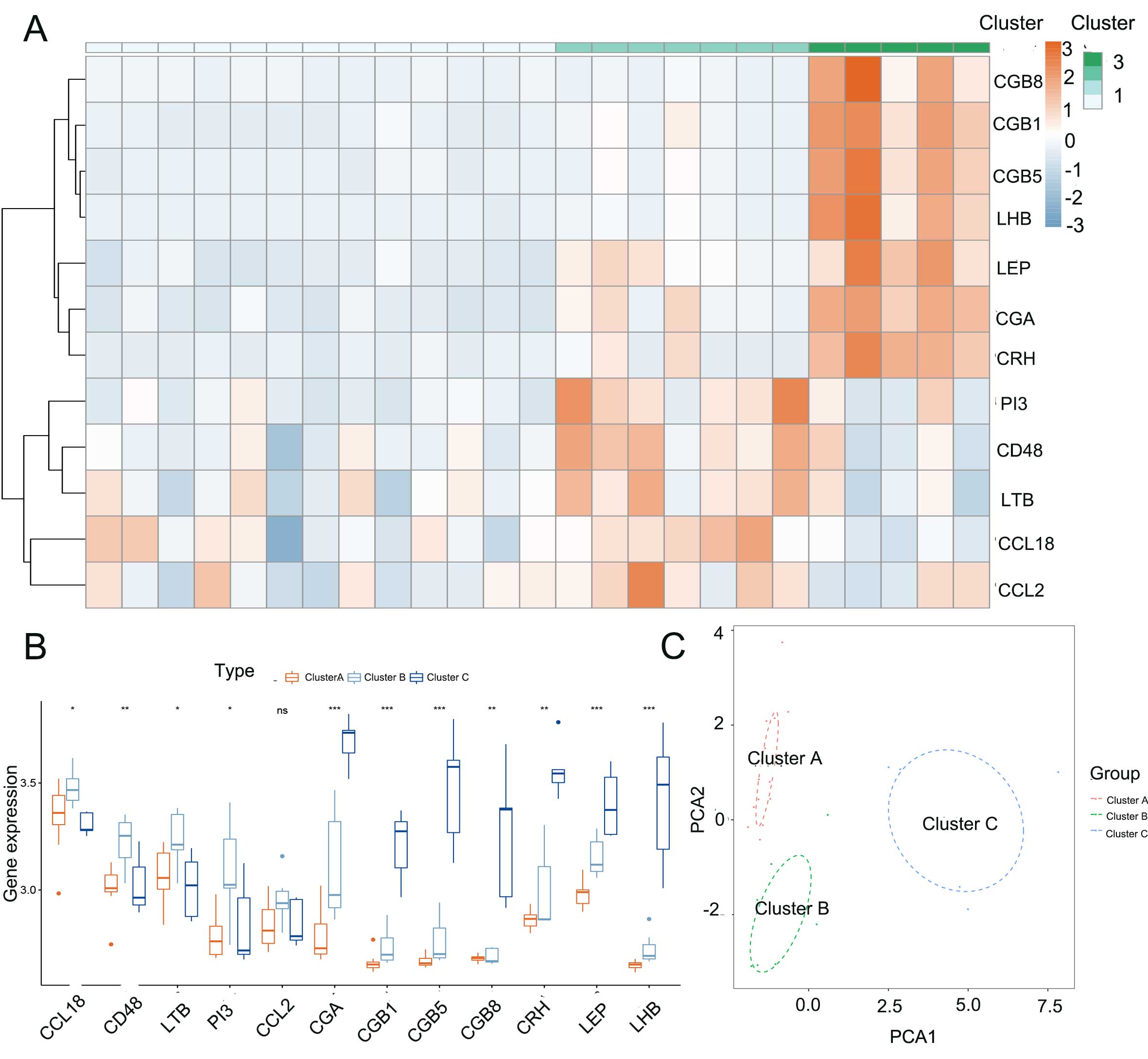

Supplement: Supplementary Figure 5 — Landscape of three subgroups in PE pregnancies in validation dataset. (A) The expression heat map of 12 DIRGs between the three subgroups. (B) The differential expression histogram of 12 DIRGs between the three subgroups. (C) Principal component analysis for the expression profiles of three subgroups that shows a remarkable difference in the transcriptomes between the different subgroups. [file Image_5.jpeg]
