# Supplementary material for: Potential of Immune-Related Genes as Biomarkers for Diagnosis and Subtype Classification of Preeclampsia
Source: Front Genet. 2020 Dec 1;11:579709. doi: 10.3389/fgene.2020.579709 (PMC7737719; doi:10.3389/fgene.2020.579709)
Supplement: Supplementary Table 1 — Thirty-six immune genes in our analysis overlapped with the original article according to the screening criteria of P < 0.05. [file Table_1.DOCX]

| gene | conMean | treatMean | logFC | pValue |
| --- | --- | --- | --- | --- |
| ADIPOR2 | 9.218517476 | 9.109929771 | -0.017094822 | 0.036430268 |
| BRAF | 6.765117333 | 6.831528229 | 0.014093392 | 0.000732115 |
| BTK | 8.728752857 | 8.429695743 | -0.05029498 | 0.023484275 |
| CCR3 | 6.863579452 | 6.697246886 | -0.035393006 | 0.001465631 |
| CCR7 | 8.123176548 | 7.665880486 | -0.083592494 | 0.011795077 |
| CD14 | 12.4695355 | 12.0536066 | -0.04894284 | 0.000676008 |
| CD19 | 6.980874476 | 6.835270429 | -0.030409353 | 0.014513515 |
| CD1D | 7.311824667 | 7.131656543 | -0.035994251 | 0.002194864 |
| CD4 | 7.92885881 | 7.706081114 | -0.041115864 | 0.019887007 |
| CD72 | 7.322433143 | 7.139703771 | -0.036458898 | 0.000349582 |
| CX3CR1 | 8.121810952 | 7.660047486 | -0.084448111 | 0.001764254 |
| CXCL5 | 7.383192548 | 7.210594571 | -0.034126557 | 0.003845249 |
| ENG | 9.553630214 | 9.921930029 | 0.054571747 | 0.040250289 |
| GPER | 7.242945048 | 7.368741629 | 0.02484184 | 0.016775157 |
| GPI | 9.623900071 | 9.423857343 | -0.030303963 | 0.006811406 |
| GPR17 | 6.955339405 | 7.022642971 | 0.013893174 | 0.012522112 |
| HCK | 9.31956019 | 8.913569457 | -0.064258595 | 0.021029362 |
| HSPA6 | 8.667124548 | 8.409768314 | -0.043487381 | 0.021029362 |
| ICAM2 | 9.937855095 | 9.710213457 | -0.033431496 | 0.012522112 |
| IFI30 | 10.12077919 | 9.778060086 | -0.04970019 | 0.045060476 |
| IL10RA | 8.441785643 | 8.189660371 | -0.043744572 | 0.024135053 |
| IL6ST | 7.325074857 | 7.562949971 | 0.046105573 | 0.006746219 |
| INSL3 | 6.731819833 | 6.6837288 | -0.01034337 | 0.033227582 |
| ITGAL | 7.908302905 | 7.567126229 | -0.063622619 | 0.010773835 |
| LEFTY1 | 7.107456095 | 7.493515114 | 0.076309346 | 0.006532917 |
| LILRB3 | 11.94912024 | 11.63640311 | -0.038259222 | 0.019336172 |
| LMBR1 | 8.132906381 | 8.2298298 | 0.017091588 | 0.021621411 |
| LTB | 8.931282952 | 8.242040943 | -0.115865799 | 0.003020314 |
| MET | 8.084828048 | 8.202994114 | 0.020933503 | 0.04618799 |
| NTF3 | 7.108888786 | 7.217754714 | 0.021926051 | 0.02480121 |
| PI3 | 8.461040238 | 7.713865943 | -0.133380972 | 0.010773835 |
| PLAUR | 8.612567476 | 8.439176371 | -0.029341176 | 0.012522112 |
| SPP1 | 12.7472524 | 12.17385569 | -0.066400147 | 0.021029362 |
| TNFRSF14 | 10.82411083 | 10.39428834 | -0.058457531 | 0.000323159 |
| TNFSF13B | 9.77980319 | 9.403403171 | -0.056622458 | 0.02480121 |
| TYK2 | 9.717411976 | 9.484961971 | -0.034930145 | 0.00592849 |
