# Supplementary material for: Potential of Immune-Related Genes as Biomarkers for Diagnosis and Subtype Classification of Preeclampsia
Source: Front Genet. 2020 Dec 1;11:579709. doi: 10.3389/fgene.2020.579709 (PMC7737719; doi:10.3389/fgene.2020.579709)
Supplement: Supplementary Table 2 — The risk score of each patients in training dataset. [file Table_2.DOCX]

| id | gestational weeks | preeclampsia | CCL18 | CCL2 | CRH | PI3 | riskScore | risk |
| --- | --- | --- | --- | --- | --- | --- | --- | --- |
| GSM1480147 | 25 | 1 | 7.25508 | 7.651346 | 11.692997 | 6.829189 | 24.39603686 | high |
| GSM1480156 | 25 | 1 | 7.645377 | 8.231277 | 10.386512 | 7.103385 | 9.659597533 | high |
| GSM1480137 | 27 | 1 | 9.587208 | 11.77947 | 7.945699 | 7.956798 | 0.168578942 | low |
| GSM1480138 | 27 | 1 | 7.194927 | 7.93432 | 11.365868 | 6.835057 | 16.62297726 | high |
| GSM1480150 | 27 | 1 | 7.662478 | 10.827592 | 7.186943 | 6.906715 | 0.366473203 | low |
| GSM1480167 | 27 | 1 | 7.723858 | 8.649339 | 13.88636 | 6.592376 | 19.41893339 | high |
| GSM1480142 | 28 | 1 | 7.288212 | 8.211328 | 9.669374 | 7.24735 | 6.956863082 | high |
| GSM1480148 | 28 | 1 | 8.220311 | 8.748482 | 7.328287 | 8.412166 | 1.649805347 | high |
| GSM1480154 | 29 | 1 | 7.844656 | 9.119348 | 10.286704 | 7.621729 | 3.107221051 | high |
| GSM1480128 | 30 | 1 | 8.394509 | 9.06468 | 7.95749 | 6.924601 | 3.189878823 | high |
| GSM1480166 | 30 | 1 | 7.727603 | 9.417462 | 11.362944 | 7.181429 | 3.614457257 | high |
| GSM1480133 | 31 | 1 | 8.172014 | 8.61292 | 9.196352 | 6.8279 | 6.661867073 | high |
| GSM1480134 | 31 | 1 | 8.537154 | 9.718936 | 6.923811 | 7.064234 | 1.248988833 | high |
| GSM1480139 | 31 | 1 | 7.827986 | 8.275298 | 6.805488 | 6.929364 | 4.562796423 | high |
| GSM1480140 | 31 | 1 | 7.912047 | 9.671047 | 10.616953 | 7.22418 | 2.417575908 | high |
| GSM1480144 | 31 | 1 | 8.118477 | 10.432835 | 6.98632 | 7.055131 | 0.549188497 | low |
| GSM1480152 | 31 | 1 | 8.70681 | 9.993208 | 6.748679 | 6.923237 | 1.027003667 | high |
| GSM1480129 | 32 | 1 | 7.708671 | 8.727823 | 7.229437 | 10.340499 | 0.517232999 | low |
| GSM1480146 | 32 | 1 | 7.915461 | 9.885283 | 6.937236 | 7.059615 | 0.887764475 | low |
| GSM1480130 | 33 | 1 | 6.981321 | 7.40552 | 9.913197 | 8.779161 | 6.823050243 | high |
| GSM1480132 | 33 | 1 | 8.938985 | 8.091307 | 9.995845 | 11.008823 | 1.903552429 | high |
| GSM1480136 | 33 | 1 | 8.361346 | 7.656597 | 7.291559 | 8.471186 | 4.972220193 | high |
| GSM1480135 | 34 | 1 | 8.180653 | 9.516865 | 6.955593 | 7.220769 | 1.282980298 | high |
| GSM1480141 | 35 | 1 | 7.487391 | 9.313342 | 7.637194 | 7.176308 | 1.552545456 | high |
| GSM1480151 | 35 | 1 | 10.070676 | 10.141443 | 10.25446 | 8.017939 | 1.695485179 | high |
| GSM1480131 | 36 | 1 | 6.87238 | 9.155361 | 14.244842 | 6.781027 | 8.992642018 | high |
| GSM1480107 | 37 | 0 | 8.560112 | 9.579879 | 6.865132 | 9.938928 | 0.316568872 | low |
| GSM1480155 | 37 | 1 | 8.578677 | 9.825329 | 6.886856 | 7.720328 | 0.798118933 | low |
| GSM1480093 | 38 | 0 | 8.892887 | 9.948393 | 7.252736 | 8.790799 | 0.480415407 | low |
| GSM1480095 | 38 | 0 | 8.957307 | 9.849839 | 7.353551 | 7.878884 | 0.892669502 | low |
| GSM1480096 | 38 | 0 | 8.668138 | 9.906963 | 8.576232 | 7.149884 | 1.517866885 | high |
| GSM1480098 | 38 | 0 | 9.626837 | 11.17929 | 7.878448 | 7.527227 | 0.3851799 | low |
| GSM1480101 | 38 | 0 | 9.073716 | 10.249833 | 7.601467 | 6.947716 | 1.064718859 | high |
| GSM1480103 | 38 | 0 | 8.831106 | 9.806353 | 6.803615 | 7.088271 | 1.194420735 | high |
| GSM1480106 | 38 | 0 | 8.957307 | 9.026181 | 7.516252 | 11.70944 | 0.286575875 | low |
| GSM1480115 | 38 | 0 | 8.086336 | 8.809985 | 7.737173 | 10.223494 | 0.636532549 | low |
| GSM1480124 | 38 | 0 | 9.548246 | 9.477921 | 8.868279 | 10.273044 | 0.629655874 | low |
| GSM1480145 | 38 | 1 | 8.640867 | 8.780951 | 8.591081 | 10.88091 | 0.667319206 | low |
| GSM1480162 | 38 | 1 | 10.6177 | 10.132268 | 8.64168 | 8.970374 | 0.829814946 | low |
| GSM1480163 | 38 | 1 | 8.450351 | 8.898335 | 6.899631 | 7.428879 | 2.290970498 | high |
| GSM1480165 | 38 | 1 | 7.766304 | 9.684047 | 7.842009 | 7.0372 | 1.306634862 | high |
| GSM1480092 | 39 | 0 | 7.382419 | 9.049399 | 6.729032 | 6.667444 | 2.069330912 | high |
| GSM1480097 | 39 | 0 | 10.826595 | 11.411358 | 6.740758 | 7.636577 | 0.310416392 | low |
| GSM1480100 | 39 | 0 | 10.432835 | 10.810692 | 6.996462 | 9.637794 | 0.189304564 | low |
| GSM1480104 | 39 | 0 | 8.894994 | 10.649121 | 6.826162 | 7.093768 | 0.520895789 | low |
| GSM1480105 | 39 | 0 | 7.666578 | 9.221933 | 6.776743 | 6.6667 | 1.909061447 | high |
| GSM1480108 | 39 | 0 | 8.691604 | 8.687868 | 7.04865 | 10.563953 | 0.609128621 | low |
| GSM1480110 | 39 | 0 | 9.807901 | 12.108349 | 6.81697 | 7.111279 | 0.153564055 | low |
| GSM1480111 | 39 | 0 | 7.776751 | 10.116957 | 7.485422 | 9.617009 | 0.201436226 | low |
| GSM1480112 | 39 | 0 | 9.602882 | 10.139967 | 8.332138 | 10.526374 | 0.252842162 | low |
| GSM1480113 | 39 | 0 | 9.333071 | 10.374962 | 6.920694 | 9.941083 | 0.179401203 | low |
| GSM1480114 | 39 | 0 | 8.650817 | 10.154293 | 6.730197 | 6.839546 | 0.893471619 | low |
| GSM1480116 | 39 | 0 | 9.525248 | 10.213746 | 7.782242 | 9.546 | 0.336627537 | low |
| GSM1480117 | 39 | 0 | 9.121515 | 9.135715 | 7.828898 | 9.282757 | 1.032618006 | high |
| GSM1480118 | 39 | 0 | 9.714871 | 10.53685 | 7.996733 | 10.214076 | 0.190262133 | low |
| GSM1480121 | 39 | 0 | 8.73486 | 8.82789 | 7.022743 | 7.390512 | 2.804785948 | high |
| GSM1480122 | 39 | 0 | 9.120044 | 10.144879 | 7.695355 | 7.214938 | 1.066424628 | high |
| GSM1480125 | 39 | 0 | 10.308678 | 9.273674 | 6.719231 | 8.538625 | 1.435234965 | high |
| GSM1480127 | 39 | 0 | 9.923966 | 10.308231 | 6.980081 | 9.746079 | 0.255545446 | low |
| GSM1480159 | 39 | 0 | 8.922932 | 10.504331 | 6.921828 | 10.128987 | 0.126818191 | low |
| GSM1480160 | 39 | 0 | 9.123454 | 10.415251 | 7.122441 | 7.202554 | 0.713888939 | low |
| GSM1480161 | 39 | 0 | 7.796963 | 9.037393 | 6.700067 | 6.97411 | 1.996230378 | high |
| GSM1480143 | 39 | 1 | 9.425886 | 10.744279 | 6.866828 | 7.598298 | 0.427435471 | low |
| GSM1480149 | 39 | 1 | 8.161076 | 9.032039 | 7.794773 | 9.070336 | 0.963945739 | high |
| GSM1480153 | 39 | 1 | 9.380151 | 9.714871 | 8.498466 | 7.731218 | 1.637841138 | high |
| GSM1480094 | 40 | 0 | 8.768768 | 9.788595 | 7.028506 | 10.261968 | 0.239010307 | low |
| GSM1480102 | 40 | 0 | 9.306288 | 10.31613 | 6.907669 | 7.631336 | 0.631397476 | low |
| GSM1480120 | 40 | 0 | 11.721649 | 11.908882 | 7.165788 | 9.204141 | 0.11830727 | low |
| GSM1480123 | 40 | 0 | 7.007614 | 8.851099 | 12.517108 | 7.813863 | 4.912488273 | high |
| GSM1480157 | 40 | 0 | 8.004129 | 9.494095 | 7.246686 | 7.264541 | 1.306615766 | high |
| GSM1480091 | 41 | 0 | 7.918073 | 9.891252 | 6.529015 | 6.777607 | 0.929192505 | high |
| GSM1480099 | 41 | 0 | 9.240628 | 11.150383 | 7.210202 | 7.873209 | 0.252637103 | low |
| GSM1480126 | 41 | 0 | 9.277388 | 9.652902 | 6.990642 | 8.212121 | 0.920212245 | low |
| GSM1480158 | 41 | 0 | 9.351985 | 11.273055 | 7.050295 | 7.970879 | 0.210843546 | low |
| GSM1480164 | 41 | 1 | 8.260694 | 10.19588 | 7.177811 | 7.057597 | 0.759548993 | low |
| GSM1480109 | 42 | 0 | 8.421612 | 10.45338 | 6.915109 | 8.183623 | 0.31960296 | low |
| GSM1480119 | 42 | 0 | 8.184828 | 9.635901 | 6.843712 | 8.10255 | 0.698975445 | low |
