# Supplementary material for: Potential of Immune-Related Genes as Biomarkers for Diagnosis and Subtype Classification of Preeclampsia
Source: Front Genet. 2020 Dec 1;11:579709. doi: 10.3389/fgene.2020.579709 (PMC7737719; doi:10.3389/fgene.2020.579709)
Supplement: Supplementary file 8 [file Table_3.DOC]

#############（连续性变量）

set.seed(1234)

# load the library

library(pacman)

p_load(DALEX,caret,tidyverse)

library(mlbench)

library(caret)

library(tidyverse)

library(DALEX)

#读取数据

# load the dataset

data=read.table("risk.txt",sep="\t",header=T,row.names=1,check.names=F)

# prepare training scheme

control <- trainControl(method="repeatedcv", number=10, repeats=3)

regr_rf <- train(diabetes~., data=data,method="rf", ntree = 100)

regr_gbm <- train(diabetes~., data=data, method="gbm")

regr_nn <- train(diabetes~., data=data,

method = "nnet",

linout = TRUE,

preProcess = c('center', 'scale'),

maxit = 500,

tuneGrid = expand.grid(size = 2, decay = 0),

trControl = trainControl(method = "none", seeds = 1))

########

######################

rt=read.table("risk.txt",sep="\t",header=T,row.names=1,check.names=F)

explainer_regr_rf <- DALEX::explain(regr_rf, label="rf",

data= rt, y = rt$diabetes)

explainer_regr_gbm <- DALEX::explain(regr_gbm, label = "gbm",

data= rt, y = rt$diabetes)

explainer_regr_nn <- DALEX::explain(regr_nn, label = "nn",

data= rt, y = rt$diabetes)

mp_regr_rf <- model_performance(explainer_regr_rf)

mp_regr_gbm <- model_performance(explainer_regr_gbm)

mp_regr_nn <- model_performance(explainer_regr_nn)

mp_regr_rf

plot(mp_regr_rf, mp_regr_nn, mp_regr_gbm)

plot(mp_regr_rf, mp_regr_nn, mp_regr_gbm, geom = "boxplot")

vi_regr_rf <- variable_importance(explainer_regr_rf, loss_function = loss_root_mean_square)

vi_regr_gbm <- variable_importance(explainer_regr_gbm, loss_function = loss_root_mean_square)

vi_regr_nn <- variable_importance(explainer_regr_nn, loss_function = loss_root_mean_square)

plot(vi_regr_rf, vi_regr_gbm, vi_regr_nn)

####################（分类变量）

library(pacman)

p_load(DALEX,caret,tidyverse,breakDown)

rt=read.table("risk.txt",sep="\t",header=T,row.names=1,check.names=F)

trainIndex <- createDataPartition(rt$preeclamptic, p = 0.6, list = FALSE, times = 1)

rtTrain <- rt[ trainIndex,]

rtTest <- rt[-trainIndex,]

classif_rf <- train(preeclamptic~., data=rtTrain, method="rf", ntree = 100, tuneLength = 1)

classif_glm <- train(preeclamptic~., data=rtTrain, method="glm", family="binomial")

classif_svm <- train(preeclamptic~., data=rtTrain, method="svmRadial", prob.model = TRUE, tuneLength = 1)

#定义求得似然估计的函数

p_fun <- function(object, newdata){predict(object, newdata=newdata, type="prob")[,2]}

rt=read.table("risk2.txt",sep="\t",header=T,row.names=1,check.names=F)

#把测试集的响应变量转化为数值

##yTest <- as.numeric(as.character(rt$diabetes))

explainer_classif_rf <- DALEX::explain(classif_rf, label = "rf",

data = rt, y = rt$diabetes,

predict_function = p_fun)

explainer_classif_glm <- DALEX::explain(classif_glm, label = "glm",

data = rt, y = rt$diabetes,

predict_function = p_fun)

explainer_classif_svm <- DALEX::explain(classif_svm, label = "svm",

data = rt, y = rt$diabetes,

predict_function = p_fun)

mp_classif_rf <- model_performance(explainer_classif_rf)

mp_classif_glm <- model_performance(explainer_classif_glm)

mp_classif_svm <- model_performance(explainer_classif_svm)

plot(mp_classif_rf, mp_classif_glm, mp_classif_svm)

plot(mp_classif_rf, mp_classif_glm, mp_classif_svm, geom = "boxplot")

vi_classif_rf <- variable_importance(explainer_classif_rf, loss_function = loss_root_mean_square)

vi_classif_glm <- variable_importance(explainer_classif_glm, loss_function = loss_root_mean_square)

vi_classif_svm <- variable_importance(explainer_classif_svm, loss_function = loss_root_mean_square)

plot(vi_classif_rf, vi_classif_glm, vi_classif_svm)

library(rms)

library(readr)

data=read.table("risk.txt",sep="\t",header=T,row.names=1,check.names=F)

data=data[c(1:(ncol(data)-9))]

##data<-as.data.frame(data)

##data$race<-factor(data$race)

##attach(data)

dd <- datadist(data)

options(datadist="dd")

fit<-lrm(preeclamptic~.,data=data,x=T,y=T)

fit

summary(fit)

nom<-nomogram(fit,fun=plogis,fun.at=c(0.99, 0.9, 0.8, 0.7, 0.6, 0.5, 0.4, 0.3,0.2,0.1),lp=F,funlabel = "preeclamptic")

plot(nom)

cal<-calibrate(fit,method='boot',B=1000)

plot(cal,xlim = c(0,1.0),ylim = c(0,1.0))

#C指数计算方法1

data$predvalue<-predict(fit)

library(ROCR)

pred<-prediction(data$predvalue,data$low)

auc<-performance(pred,"auc")

auc

#C指数计算方法2

library(Hmisc)

somers2(data$predvalue,data$low)

#C指数计算方法3

v<-validate(fit,method = "boot",B=1000,dxy=T)

Dxy=v[rownames(v)=="Dxy",colnames(v)=="index.orig"]

bias_corrected_c_index<-abs(Dxy)/2+0.5

orig_c_index<-abs(orig-Dxy)/2+0.5

orig_c_index

bias_corrected_c_index

#C指数计算方法4

c<-rcorrcens(low~predict(fit,newdata=data),data=data)

c[1,1]

c[1,1]-1.96*c[1,4]/2

c[1,1]-1.96*c[1,4]/2

C指数计算方法5

fit

library(devtools)

install_github("mdbrown/DecisionCurve")

library(rmda)

setwd('D:\\DCA')

rt=read.table("risk.txt",sep="\t",header=T,row.names=1,check.names=F)

##stage<-decision_curve( fustat ~stage,data=rt,family=binomial(link='logit'),

## thresholds= seq(0,1, by = 0.01),

## confidence.intervals = 0.95,

## study.design = 'case-control',

CCL18<-decision_curve(preeclamptic ~ CCL18, data=rt,family=binomial(link='logit'),

bootstraps = 50)

CCL2<-decision_curve(preeclamptic ~ CCL2, data=rt,family=binomial(link='logit'),

bootstraps = 50)

PI3<-decision_curve(preeclamptic ~PI3, data=rt,family=binomial(link='logit'),

bootstraps = 50)

CRH<-decision_curve(preeclamptic ~ CRH, data=rt,family=binomial(link='logit'),

bootstraps = 50)

nomogram<-decision_curve(preeclamptic ~ CCL18+CCL2+PI3+ CRH, data=rt,family=binomial(link='logit'),

bootstraps = 50)

plot_decision_curve(list(CCL18,CCL2,PI3, CRH,nomogram),

curve.names=c('CCL18','CCL2','PI3','CRH','nomogram'),

confidence.intervals = FALSE,

cost.benefit.axis = TRUE,

xlim=c(0,1),

lwd = 1.2,

)

##绘制临床影响曲线（Clinical Impact Curve）

plot_clinical_impact(stage,population.size= 1000,

cost.benefit.axis = T,

n.cost.benefits= 8,

col =c('red','blue'),

confidence.intervals= T,

ylim=c(0,1000),

legend.position="topright")

plot_clinical_impact(nomogram,population.size= 1000,

cost.benefit.axis = T,

n.cost.benefits= 8,col =c("#6D9EC1", "#E46726"),

confidence.intervals=T,

ylim=c(0,1000),

legend.position="topright")

###Video source: http://study.163.com/provider/1026136977/index.htm?share=2&shareId=1026136977

######Video source: http://www.biowolf.cn/shop/

######??????ѧ??: http://www.biowolf.cn/

######???????䣺2749657388@qq.com

######????΢??: 18520221056

##R语言机器学习：mlr包的使用和模型解释（离散变量）

library(pacman)

p_load(DALEX,breakDown,mlr)

rt=read.table("risk.txt",sep="\t",header=T,row.names=1,check.names=F)

train_index <- sample(1:nrow(rt), 0.6 * nrow(rt))

test_index <- setdiff(1:nrow(rt), train_index)

rtTest <- rt[test_index,]

#问题定义：这是一个分类模型，响应变量为quality

classif_task <- makeClassifTask(id = "ap", data = rt, target = "preeclamptic")

#模型定义：注意返回值我们要返回极大似然估计

classif_lrn_rf <- makeLearner("classif.randomForest", predict.type = "prob")

classif_lrn_glm <- makeLearner("classif.binomial", predict.type = "prob")

classif_lrn_svm <- makeLearner("classif.ksvm", predict.type = "prob")

#训练模型：注意要写清楚子集，即训练集的行号

classif_rf <- train(classif_lrn_rf, classif_task, subset=train_index)

classif_glm <- train(classif_lrn_glm, classif_task, subset=train_index)

classif_svm <- train(classif_lrn_svm, classif_task, subset=train_index)

y_test <- as.numeric(as.character(rtTest$preeclamptic))

custom_predict_classif <- function(object, newdata) {pred <- predict(object, newdata=newdata)

response <- pred$data[,3]

return(response)}

explainer_classif_rf <- DALEX::explain(classif_rf, data=rtTest, y=y_test, label= "rf", predict_function = custom_predict_classif)

explainer_classif_glm <- DALEX::explain(classif_glm, data=rtTest, y=y_test, label="glm", predict_function = custom_predict_classif)

explainer_classif_svm <- DALEX::explain(classif_svm, data=rtTest, y=y_test, label ="svm", predict_function = custom_predict_classif)

mp_classif_rf <- model_performance(explainer_classif_rf)

mp_classif_glm <- model_performance(explainer_classif_glm)

mp_classif_svm <- model_performance(explainer_classif_svm)

plot(mp_classif_rf, mp_classif_glm, mp_classif_svm)

plot(mp_classif_rf, mp_classif_glm, mp_classif_svm, geom = "boxplot")

vi_classif_rf <- variable_importance(explainer_classif_rf, loss_function = loss_root_mean_square)

vi_classif_glm <- variable_importance(explainer_classif_glm, loss_function = loss_root_mean_square)

vi_classif_svm <- variable_importance(explainer_classif_svm, loss_function = loss_root_mean_square)

plot(vi_classif_rf, vi_classif_glm, vi_classif_svm)

#####################RF

library(pacman)

p_load(DALEX,caret,tidyverse,breakDown)

rt=read.table("risk.txt",sep="\t",header=T,row.names=1,check.names=F)

trainIndex <- createDataPartition(rt$preeclamptic, p = 0.6, list = FALSE, times = 1)

rtTrain <- rt[ trainIndex,]

rtTest <- rt[-trainIndex,]

library(randomForest)

#随机森林计算（默认生成 500 棵决策树），详情 ?randomForest

set.seed(123)

rtTrain.forest <- randomForest(preeclamptic~., data = rtTrain, importance = TRUE)

rtTrain.forest

##OTU 的重要性评估

#查看表示每个预测变量（细菌 OTU）重要性的得分

#

summary(rtTrain.forest)

importance_genes <- rtTrain.forest$importance

head(importance_genes)

#或者使用函数 importance()

importance_genes <- data.frame(importance(rtTrain.forest), check.names = FALSE)

head(importance_genes)

#作图展示 top30 重要的 OTUs

varImpPlot(rtTrain.forest, n.var = min(30, nrow(rtTrain.forest$importance)),

main = 'variable importance')

importance_genes <- importance_genes[order(importance_genes$IncNodePurity, decreasing = TRUE), ]

head(importance_genes)

#输出表格

write.table(importance_genes, 'importance_genes.txt', sep = '\t', col.names = NA, quote = FALSE)

##交叉验证辅助评估选择特定数量的 OTU

#5 次重复十折交叉验证

set.seed(123)

rtTrain.cv <- replicate(5, rfcv(rtTrain[-ncol(rtTrain)], rtTrain$preeclamptic, cv.fold = 10, step = 1.5), simplify = FALSE)

rtTrain.cv

#提取验证结果绘图

rtTrain.cv <- data.frame(sapply(rtTrain.cv, '[[', 'error.cv'))

rtTrain.cv$genes <- rownames(rtTrain.cv)

rtTrain.cv <- reshape2::melt(rtTrain.cv, id = 'genes')

rtTrain.cv$genes <- as.numeric(as.character(rtTrain.cv$genes))

rtTrain.cv.mean <- aggregate(rtTrain.cv$value, by = list(rtTrain.cv$genes), FUN = mean)

head(rtTrain.cv.mean, 10)

#拟合线图

library(ggplot2)

ggplot(rtTrain.cv.mean, aes(Group.1, x)) +

geom_line() +

theme(panel.grid = element_blank(), panel.background = element_rect(color = 'black', fill = 'transparent')) +

labs(title = '',x = 'Number of genes', y = 'Cross-validation error')

##分类器

#加载 R 包

library(pROC)

library(ggplot2)

data=read.table("risk.txt",header=T,sep="\t",row.names=1)

roc6 <-roc(data$preeclamptic,data$sum)

plot(roc6,plot=TRUE, print.thres=TRUE, print.auc=TRUE,col="red")

##############################rf2

#####################RF

library(pacman)

p_load(DALEX,caret,tidyverse,breakDown)

rt=read.table("risk.txt",sep="\t",header=T,row.names=1,check.names=F)

trainIndex <- createDataPartition(rt$preeclamptic, p = 0.6, list = FALSE, times = 1)

rtTrain <- rt[ trainIndex,]

rtTest <- rt[-trainIndex,]

#randomForest 包的随机森林

library(randomForest)

#

##关键 OTUs 识别

#查看表示每个变量（OTUs）重要性的得分

#summary(otu_train.forest)

importance_genes <- rttrain.forest$importance

head(importance_genes)

#或者使用函数 importance()

importance_genes<- data.frame(importance(rttrain.forest))

head(importance_genes)

#可以根据某种重要性的高低排个序，例如根据“Mean Decrease Accuracy”指标

importance_genes <- importance_genes[order(importance_genes$MeanDecreaseAccuracy, decreasing = TRUE), ]

head(importance_genes)

#输出表格

write.table(importance_genes, 'importance_genes.txt', sep = '\t', col.names = NA, quote = FALSE)

varImpPlot(rtTrain.forest, n.var = min(30, nrow(rtTrain.forest$importance)), main = 'variable importance')

##交叉验证帮助选择特定数量的 OTUs

#5 次重复十折交叉验证

set.seed(123)

rtTrain.cv <- replicate(5, rfcv(rtTrain[-ncol(rtTrain)], rtTrain$preeclamptic, cv.fold = 10,step = 1.5), simplify = FALSE)

rtTrain.cv

#提取验证结果绘图

rtTrain.cv <- data.frame(sapply(rtTrain.cv, '[[', 'error.cv'))

rtTrain.cv$genes <- rownames(rtTrain.cv)

rtTrain.cv <- reshape2::melt(rtTrain.cv, id = 'genes')

rtTrain.cv$genes <- as.numeric(as.character(rtTrain.cv$genes))

#拟合线图

library(ggplot2)

library(splines) #用于在 geom_smooth() 中添加拟合线，或者使用 geom_line() 替代 geom_smooth() 绘制普通折线

p <- ggplot(rtTrain.cv, aes(genes, value)) +

geom_smooth(se = FALSE, method = 'glm', formula = y~ns(x, 6)) +

theme(panel.grid = element_blank(), panel.background = element_rect(color = 'black', fill = 'transparent')) +

labs(title = '',x = 'Number ofgenes', y = 'Cross-validation error')

p

#######################consensus clustering

#if (!requireNamespace("BiocManager", quietly = TRUE))

# install.packages("BiocManager")

#BiocManager::install("ConsensusClusterPlus")

#if (!requireNamespace("BiocManager", quietly = TRUE))

# install.packages("BiocManager")

#BiocManager::install("limma")

library(limma)

workDir="F:\\WANGYING2\\68m6A\\68m6A\\10.ConsensusClusterPlus" #????Ŀ¼

setwd(workDir)

rt=read.table("m6Aexp.txt",sep="\t",header=T,check.names=F) #??ȡ?????ļ?

#һ?????????ֶ??У?ȡ??ֵ

rt=as.matrix(rt)

rownames(rt)=rt[,1]

exp=rt[,2:ncol(rt)]

dimnames=list(rownames(exp),colnames(exp))

data=matrix(as.numeric(as.matrix(exp)),nrow=nrow(exp),dimnames=dimnames)

data=avereps(data)

data=data[rowMeans(data)>0,]

#ɾ????????Ʒ

group=sapply(strsplit(colnames(data),"\\-"),"[",4)

group=sapply(strsplit(group,""),"[",1)

group=gsub("2","1",group)

data=data[,group==0]

#????

maxK=9

library(ConsensusClusterPlus)

results = ConsensusClusterPlus(data,

maxK=maxK,

reps=50,

pItem=0.8,

pFeature=1,

title=workDir,

clusterAlg="km",

distance="euclidean",

seed=123456,

plot="png")

#????????

clusterNum=2 #?ּ??࣬?????жϱ?׼?ж?

cluster=results[[clusterNum]][["consensusClass"]]

write.table(cluster,file="cluster.txt",sep="\t",quote=F,col.names=F)
