# Supplementary material for: Potential of Immune-Related Genes as Biomarkers for Diagnosis and Subtype Classification of Preeclampsia
Source: Front Genet. 2020 Dec 1;11:579709. doi: 10.3389/fgene.2020.579709 (PMC7737719; doi:10.3389/fgene.2020.579709)
Supplement: Supplementary file 9 [file Table_4.DOCX]

| gene | conMean | treatMean | logFC | pValue |
| --- | --- | --- | --- | --- |
| PI3 | 8.461040238 | 7.713865943 | -0.133380972 | 0.010773835 |
| CCL18 | 8.994379333 | 8.217637057 | -0.130300117 | 0.000117301 |
| CCL2 | 10.03269452 | 9.2354408 | -0.119456402 | 0.00038111 |
| LTB | 8.931282952 | 8.242040943 | -0.115865799 | 0.003020314 |
| CD48 | 8.995392667 | 8.347016 | -0.107925723 | 0.007191443 |
| LEP | 7.826670667 | 8.457163829 | 0.111775186 | 0.022848593 |
| CGB1 | 7.049239333 | 7.663671343 | 0.120568104 | 0.003469714 |
| GDF15 | 8.363766381 | 9.315053171 | 0.15541124 | 0.01629984 |
| LHB | 7.08780431 | 7.928629086 | 0.161732663 | 0.011445623 |
| CGB8 | 7.176896333 | 8.174694886 | 0.187804802 | 0.021621411 |
| CGA | 8.495501333 | 9.765370743 | 0.200975731 | 0.014094512 |
| CGB5 | 7.144444048 | 8.332543886 | 0.22193526 | 0.002358258 |
| CRH | 7.35786819 | 8.743007971 | 0.248841882 | 0.000350388 |
